# Cyclic Fatigue Comparison of TruNatomy, Twisted File, and ProTaper Next Rotary Systems

**DOI:** 10.1155/2020/3190938

**Published:** 2020-02-26

**Authors:** Abdullah Mahmoud Riyahi, Amr Bashiri, Khalid Alshahrani, Saad Alshahrani, Hadi M. Alamri, Dina Al-Sudani

**Affiliations:** ^1^Department of Restorative Dental Sciences, Division of Endodontics, College of Dentistry, King Saud University, P.O. Box 60169, Riyadh 11545, Saudi Arabia; ^2^College of Dentistry, King Saud University, P.O. Box 60169, Riyadh 11545, Saudi Arabia; ^3^Department of Conservative Dental Sciences, College of Dentistry, Prince Sattam bin Abdulaziz University, Al-kharj 11942, Saudi Arabia; ^4^Department of Restorative Dental Sciences, College of Dentistry, King Saud University, P.O. Box 60169, Riyadh 11545, Saudi Arabia

## Abstract

TruNatomy (TN; Dentsply Sirona, Maillefer, Ballaigues, Switzerland) is a newly released system that was not tested in any previous studies. The objective of this work is to evaluate cyclic fatigue resistance of the new file and compare it with the Twisted Files (TF) and ProTaper Next (PTN). Forty-five files were distributed into 3 groups: PTN X2 (size 25 and taper 0.06), TF (size 25 and taper 0.06), and TN prime file (size 26 and taper 0.04). Each group included 15 files. Lengths of all files were 25 mm. Cyclic fatigue testing was done using artificial stainless-steel canals with 60-degree curvature and 5 mm radius. Continuous rotation movement at 300 rpm was used until the file fractures. Time for file separation was recorded in seconds. The number of cycles to failure (NCF) mean and standard deviation for each group was calculated. For statistical analysis of data, ANOVA and Tukey's multiple comparison test were used. Mean and standard deviation (SD) of NCF were 259 ± 37.2, 521.67 ± 63.07 and 846.67 ± 37.16 for PTN, TF, and TN respectively. TN on average had significantly the highest NCF compared with PTN (*p* < 0.05) and TF (*p* < 0.05). TruNatomy file showed superior cyclic fatigue resistance. With its potential to preserve tooth structure, this file offers a good cyclic fatigue advantage. However, future studies are required to evaluate other properties of this file and to examine its clinical performance.

## 1. Introduction

One of the primary concerns in the practice of endodontics is the separation of rotary instrument as a result of cyclic fatigue, particularly in root canals with severe curvatures. The nickel-titanium (NiTi) instrument cyclic fatigue is influenced by various factors [1].

Because resistance to fracture is a vital criterion of rotary NiTi instruments, manufacturers have kept a continuous effort to enhance the resistance to cyclic fatigue of (NiTi) files mainly via thermomechanical processing, mechanical design, and meticulous raw material selection [2–4].

Focusing on metallurgy, a manufacturing technique was established to develop the NiTi M-Wire. This technique utilizes thermal treatment to enhance cyclic fatigue resistance and flexibility of the instrument. ProTaper Next (PTN; Dentsply Maillefer, Ballaigues, Switzerland) is an M-Wire rotary file system that is produced by heat treatment which is reported to enhance the cyclic flexibility and fatigue resistance [5, 6]. The file has an off-centered rectangular cross section that enhances canal preparation efficiency. The system contains five different sizes (X1–X5).

A different manufacturer, SybronEndo (Orange, CA), used thermal treatment with twisting (instead of grinding) of NiTi wires to develop the Twisted File (TF). TF (SybronEndo, Orange, CA) is made by a twisting process that is mentioned to have more fracture resistance compared with ground instruments [2, 7]. The R-phase technology is used in processing of these files. The manufacturer claims superior flexibility and cyclic fatigue resistance [8].

TruNatomy (TN; Dentsply Sirona, Maillefer, Ballaigu/es, Switzerland) is a newly released file system manufactured from a 0.8 mm NiTi wire instead of 1.2 mm NiTi wire that is used to manufacture most generic files, followed by a special heat treatment. It is claimed that TruNatomy has a reduced risk of separation due to the increased resistance to cyclic fatigue and flexibility [9].

The aim of this work is to assess the cyclic fatigue resistance of this newly released system and to compare it with familiar rotary file systems (ProTaper Next and Twisted Files).

## 2. Materials and Methods

Forty-five NiTi files were distributed into 3 different groups (*n* = 15 per group). Group 1 consists of PTN X2 with tip size 25 and 0.06 taper, group 2 consists of TF instrument with tip size 25 and 0.06 taper, and group 3 consists of TN Prime File with tip size 26 and 0.04 taper. All files have the same length (25 mm). An artificial stainless-steel canal was used to test cyclic fatigue. The artificial canal had a 60-degree curvature with 5 mm radius. The center of the curvature was 6 mm from the instrument tip. The canal was sealed with a glass cover to prevent instrument slippage [10] and to secure the separated part to be retrieved. Furthermore, the device has a custom-made attachment for the handpiece that can be adjusted in the vertical and horizontal planes to provide the desired position. The device is attached to a base that allows three-dimensional reproducible position.

X-Smart rotary endodontic motor (X-Smart; Dentsply Maillefer) with a contra angle (16 : 1 reduction) was attached to the device. Every file was rotated at 300 rpm rotational speed until it is separated inside the artificial stainless-steel canal. The required time for separation was recorded in seconds. Afterwards, the number of cycles to fracture (NCF) was calculated by multiplying the time required (in seconds) to failure by rotational speed [11]. A single operator conducted the experiment.

### 2.1. Statistical Analysis

IBM-SPSS Statistics for Windows, version 25 software (Armonk, NY: IBM Corp.) was used. NCF mean and standard deviation for each group were calculated. ANOVA followed by Tukey's multiple comparison test was utilized to analyze and compare the means. A *p* value of less than 0.05 is considered statistically significant.

## 3. Results

Mean values ± SD expressed are related to the NCF. A higher number of cycles to fracture reflect the higher cyclic fatigue resistance of the tested instruments. The mean values and SD of NCF for each group are shown in Table 1.

Furthermore, ANOVA showed a statistically significant difference (*p* < 0.05). Tukey's multiple comparison test (Table 1) showed that PTN had the least NCF compared with TF (*p* < 0.05) and TN (*p* < 0.05). Moreover, TN on average had the highest NCF compared with PTN (*p* < 0.05) and TF (*p* < 0.05).

## 4. Discussion

Rotary instrument intracanal separation could be a serious mishap since the separated segments can limit the irrigation solution access to the canal system that may result in preventing sufficient microorganism elimination [12]. Therefore, comparing the file resistance to cyclic failure is an important subject that was heavily discussed in the literature [13–15].

The aim of this work is to compare fatigue resistance of the newly introduced TN files system that is made using special heat-treated NiTi with other common rotary systems in the market.

In the present study, we have tested a new brand TN system to determine if the new advancement in NiTi instrument improves file quality which could possibly improve instrument resistance to cyclic fatigue. The design characteristics of the TN file includes a special heat-treated wire, uses a 0.8 mm NiTi wire instead of 1.2 mm NiTi wire, and is operated at a higher speed. One of the claimed advantages of the TN files is that it has a reduced risk of separation due to the increased flexibility and cyclic fatigue resistance. The Prime file that was used in our experiment has a tip size of 26 with a 0.04 taper. Prime TN was compared with the PTN X2 (size 25 and taper 0.06) knowing it has a different taper. However, other reports in the literature have also compared the cyclic fatigue resistance of files with different tapers [16]. The main goal of this experiment is to test the performance of different file systems in term of resistance to cyclic fatigue.

In this study, PTN exhibited the least NCF. When compared with TF, the results could be related to the R-phase NiTi alloy from which TF is manufactured and the twisting design that eliminates the propagation of cracks produced by the traditional grinding technique, this is in consonance with the views of other studies [17–19].

The TN files exhibited the greatest mean NCF compared with the two other groups. This could be attributed to several file characteristics; the thermomechanical processing for the TruNatomy as indicated by the manufacturer is made of a special heat treatment NiTi wire, which could have played a role in improving their number of cycles to failure (NCF). However, they have not disclosed any details about the type of NiTi wire used [19, 20].

TruNatomy shaping file has an off-centered parallelogram cross-sectional design [21]; it might be speculated that this design compared with PTN rectangular cross section and the TF equilateral triangular cross section could contribute to the higher cyclic fatigue resistance of the TN files.

In addition, the fact that the TruNatomy file is made of a thin NiTi wire (0.8 mm) might have resulted in increasing cyclic fatigue resistance [22].

## 5. Conclusion

The TruNatomy system has a superior cyclic fatigue resistance compared with the TF and PTN file systems. TF is significantly more resistant to fatigue compared with PTN.

With TruNatomy potential to preserve tooth structure, this file has an advantage of enhanced cyclic fatigue resistance. However, future studies are required to evaluate other properties of this file and to examine its clinical performance.

## Figures and Tables

**Table 1:** Mean and SD of NCF of the three compared groups. ANOVA showed a significant *p* value (*p* < 0.05). Tukey's multiple comparison test results are presented.

Group	Number	Mean (NCF)	SD	ANOVA *p* value	95% confidence interval for mean		Multiple comparison test		
					Lower bound	Upper bound	NCF PN	NCF TF	NCF TN
PTN	15	259.00	37.235	0.000	238.38	279.62	1		
TF	15	521.67	63.066		486.74	556.59	0.000	1	
TN	15	846.67	37.161		826.09	867.25	0.000	0.000	1

## Data Availability

The data used to support the findings of this study are included within the article.
